# Dataset for a Norwegian high-voltage distribution system (132/66 kV)

**DOI:** 10.1016/j.dib.2026.113333

**Published:** 2026-10-09

**Authors:** Susanne Sandell, Sigurd Hofsmo Jakobsen, Astrid Lye Moum, Daniel Bjerkehagen, Harald G Svendsen

**Affiliations:** SINTEF Energy Research, Postboks 4761 Torgarden, Trondheim, 7465, Norway

**Keywords:** Regional distribution grid, Realistic grid dataset, Load and generation time series, Risk-based grid operation, Grid planning with active measures

## Abstract

This article presents a dataset representing a Norwegian high-voltage (HV) distribution system operated at 132/66 kV. The dataset was mainly developed in the Norwegian research centre SecurEL and will in brief be referred to as the “SecurEL reference system”. Data from a real Norwegian HV distribution system provided by a distribution grid company and was anonymized and processed. The result is a simplified reference system which has features typical for Norwegian HV distribution systems: The grid has a mixed ring and radial topology and comprises four main power transfer corridors. The generation is dominated by reservoir hydropower. To our knowledge, this is the first openly available dataset representing this set of conditions for a Norwegian (HV) distribution grid. The grid model was anonymized by substituting the original line types with publicly available data. Load time series were generated by anonymizing original load time series by removing the temperature-dependent component, modelling the residual short-term variability as a stochastic process, and reintroducing the temperature-dependent component using publicly available weather data. The generation time series are made from a market model which takes grid constraints, demand and historic weather data into account. The load and generation data resolution is hourly, and the duration is 4 years (2019-2022).

The dataset includes historical reliability data (failure rates), load development scenarios reflecting different assumptions on future demand growth and ambient-adjusted line ratings (AAR).

The dataset can be reused for a wide range of power system analyses, including reliability studies, testing and demonstration of risk-based operational and planning methods, and evaluation of active operational measures.

Specifications Table

**Table:** 

Subject	Engineering & Materials science
Specific subject area	Power system datasets for high-voltage distribution grid analysis
Type of data	Tables, Figures Processed
Data collection	The raw grid topology was obtained from the distribution system operator’s (DSO’s) network information systems (NIS). The raw historical load data were obtained from the DSO’s operational measurement databases, populated by measurements from the supervisory control and data acquisition (SCADA) system. The temperature data was downloaded from the Norwegian Meteorological Institute’s weather database [1] Due to Norwegian legislation on sensitive power system information, the raw dataset had to be anonymized and processed. The data processing procedure is described in detail in the *Experimental Design, Materials, and Methods* section.
Data source location	Primary data source: Country: Norway. The exact location of the grid cannot be disclosed.
Data accessibility	Repository name: Dataset for a Norwegian high-voltage distribution system (132/66 kV) Data identification number: 10.5281/zenodo.22892065 Direct URL to data: https://doi.org/10.5281/zenodo.22892065 Related code: A companion open-source repository loads this dataset and reproduces a base-case AC power flow: https://gitlab.sintef.no/securel/securel-wp2/securel-hv-grid
Related research article	S. Sandell, I.B. Sperstad, Evaluating grid development strategies for a regional grid using dynamic line rating sensors, IEEE PES ISGT Europe, (2024). https://doi.org/10.1109/ISGTEUROPE62998.2024.10863552 [2] I.B. Sperstad, S. Sandell, S.H. Jakobsen, M. Istad, V.V. Vadlamudi, Risk-based grid planning and operation: Framework and application to hosting capacity assessment, SSRN (preprint), 2026. https://doi.org/10.2139/ssrn.6463905. [3]

## 1 Value of the Data

• Why are these data useful?
  ○ The dataset provides a realistic, anonymized representation of a Norwegian 132/66 kV HV distribution grid, including topology, electrical parameters, aggregated load and generation time series, failure rates and load development scenarios suitable for grid planning and operation method development and case studies.
• Who can benefit from these data?
  ○ Researchers, students, technology developers and distribution system operators working with grid operation, planning, and reliability assessment can benefit from the dataset.
• How can these data be used for further insights and development of experiments?
  ○ The data can be used to develop, test, and compare methods for grid planning, hosting capacity assessments, contingency analysis and operational strategies under different load development scenarios.
• What is the additional value of these data?
  ○ The dataset includes multiple power transfer corridors. This allows analyses of security of supply and contingency handling in meshed power grids. The dataset also supports static and ambient-adjusted line ratings (AAR), enabling studies of capacity utilization, cost-benefit analyses of line temperature uprating and how temperature affects grid operation.
• The grid’s nodes can be replaced or extended with detailed medium voltage (MV) or low voltage (LV) grid models, allowing multi-scale studies and integration with other synthetic grid datasets.

## 2 Background

The raw dataset was first collected in the research centre FME CINELDI (2016-2024) to support method development for power system planning and analyses a of Norwegian high-voltage distribution grid. It was developed within a power system modelling context where grid topology and electrical properties are combined with time-varying inputs for load demand and generation, and where e.g. failure statistics are required to perform reliability analysis necessary for risk-based planning and operation. Since the dataset was originally used alongside dynamic line rating sensor data, AAR were included to support studies that represent weather-dependent capacity variation. In the associated research articles, an earlier, non-anonymized version of the dataset was used in a case study to illustrate the implications of using sensor data and/or reduced operational margins in grid planning [2,3].

The motivation for publishing the dataset in the research centre FME SecurEL (2025-2032) is to support research related to power system planning and operation in a Norwegian context. The dataset describes a topology and conditions which are representative for Norwegian regional distribution grids, such as:

• Weakly meshed grid
• Temperature-sensitive load demand, since space heating is mainly electric
• Power production dominated by reservoir-based hydropower plants and some run-of-river hydropower
• Strained power-transfer corridors which were built decades ago and are now being challenged by the impending electrification of the energy system
• Increased availability of sensor equipment for temperature-dependent component ratings

Established open power grid datasets do not represent the above set of conditions. Other Norwegian reference grids represent some of the characteristics (similar load demand) but represent low- and medium voltage grids [4,5]. The SecurEL reference system is intended to address this gap, and is to the authors’ knowledge the only openly published Norwegian HV reference system. The raw dataset originally collected was anonymized and processed to meet Norwegian legal requirements for sensitive power system data. The public version of the dataset is in brief referred to as the SecurEL reference system.

## 3 Data Description

This section describes the contents and structure of the dataset for the SecurEL reference system. The dataset is provided as a grid model, time series for load and generation, additional grid information, AAR and reliability data. In all the tables, consistent identifiers for buses (nodes), branches (lines), and associated data are used.

The data description is organized in four main chapters:

1. The grid data: the grid topology and electrical parameters on MATPOWER format, as well as additional information about the grid.
2. Load data: hourly timeseries (2019-2022) for the loads in the grid.
3. Generation data: hourly timeseries (2019-2022) for the power plants in the grid.
4. Power transfer corridors: Net load duration curves for the four strained power transfer corridors in the grid based on load and generation data in the dataset.
5. Additional information: This section contains various supplementary information such as extra information about the grid components, AAR data, reliability data and temperature data.

The following subsections describe each part in detail, including file formats, key fields, and the relationships between the different data tables.

### 3.1 Grid data

Table 1 gives an overview of the files in the grid data part of the dataset.

**Table 1:** Overview of grid dataset.

File name	Description of data
SecurEL_reference_grid_base_bus.csv	Bus data on the MATPOWER case format [6] including load data and the solved AC power-flow voltage (Vm, Va) for the reference case
SecurEL_reference_grid_base_bus_extra.csv	Extra data fields for the buses in the grid which describes the placement of loads and generators in the grid.
SecurEL_reference_grid_base_branch.csv	Branch data on the MATPOWER case format
SecurEL_reference_grid_base_branch_extra.csv	Extra data fields for the branch data, in addition to those defined in the standard MATPOWER case format: length, line type, voltage, component type, installation year and type of location
SecurEL_reference_grid_base_branch_pf_sol.csv	AC power-flow solution for the base reference system data.
SecurEL_reference_grid_base_gen*.*csv	Generator data on the MATPOWER case format
SecurEL_reference_grid_base_gen*_*extra.csv	Extra data fields for the generators
standard_overhead_line_types.csv	Technical data for overhead lines in the reference system
standard_cable_types.csv	Technical data for underground cables in the reference system

Fig. 1 shows an overview of the SecurEL reference system. It is a weakly meshed, high-voltage (132/66 kV) distribution grid, also sometimes referred to as a sub-transmission grid. In Norwegian it is referred to as a regional distribution grid or regional grid. It contains 17 nodes, 20 distribution lines and 1 double transformer. The lines are mostly overhead lines operated at 132 kV as well as some underground cabled at 66 kV.

**Fig. 1:**
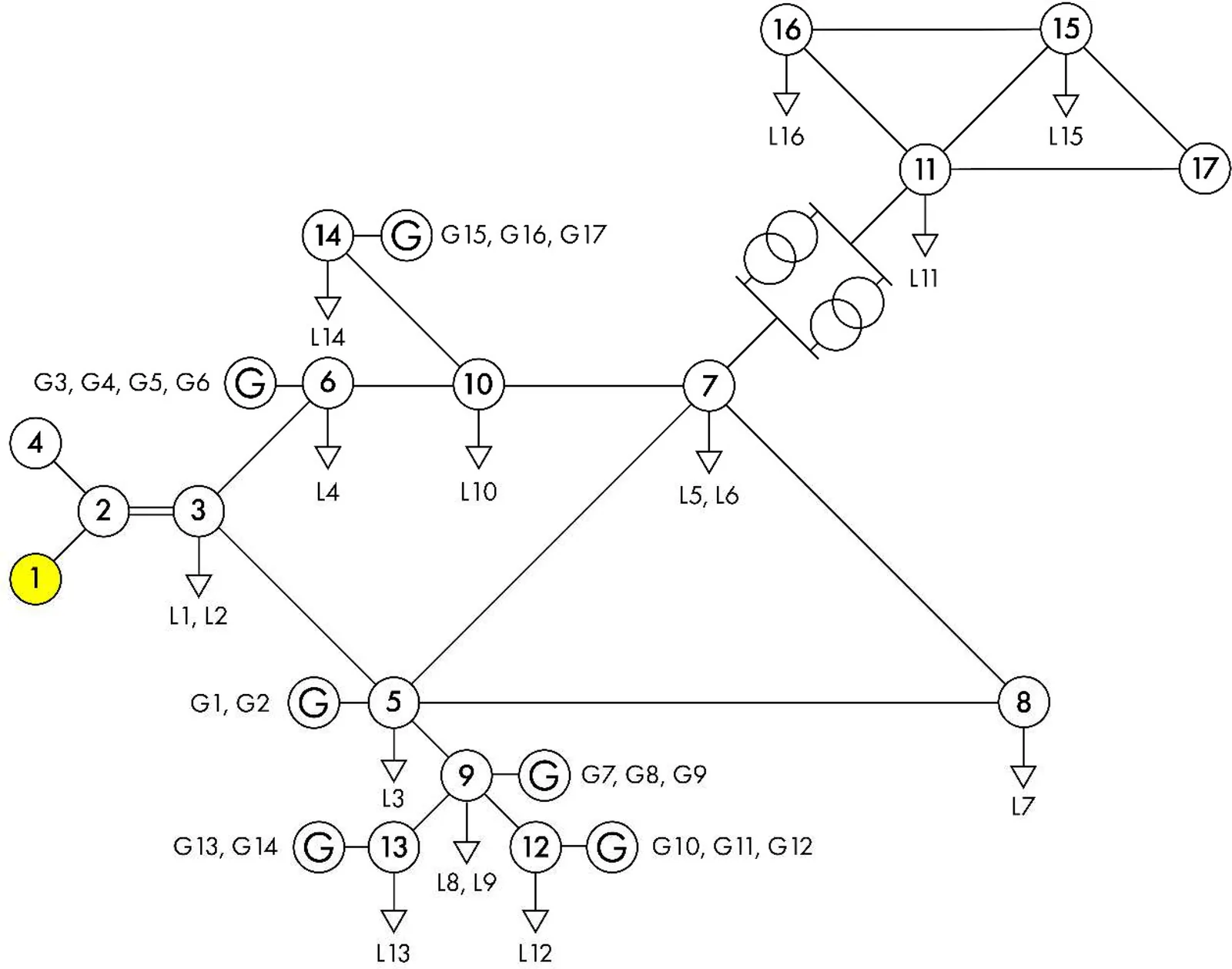
The SecurEL reference system with an extra high voltage (EHV)/HV substation (bus 1, colored yellow) and one reserve connection (bus 12).

The extra high voltage (EHV)/HV substation is located at node 1, and the reserve connection is located at node 12. The EHV/HV connects the grid to the transmission grid (420 kV), and the reserve connection is connected to a neighbouring HV distribution grid at 132 kV (not included). The EHV/HV transformers are not included in the dataset. The transformers connecting the 132 kV grid to the 66 kV grid is included and is visible as a double transformer between bus 7 and 11 in Fig. 1. Information about the reference system dataset is summarized in Table 2.

**Table 2:** A summary of the main characteristics of the SecurEL reference system.

Parameter	Value
Number of buses	17
Voltage levels	132 kV / 66 kV
Base power	10 MVA
Number of loads	16
Number of generators	17
Total peak net load (real power)	174 MW
Peak net load hour (Reference case)	2020-03-04 18:00:00
Power factor for loads	0.95

The 10 MVA base power reported in Table 2 was inherited from the MATPOWER/pandapower tooling convention used when the dataset was first produced, and is kept to avoid discrepancies with earlier versions of the dataset.

Table 3 shows an excerpt from the file SecurEL_reference_grid_base_bus.csv describing the grid’s buses, or nodes. Below the table follows an explanation of the different columns.

**Table 3:** Example of bus data from SecurEL_reference_grid_base_bus.csv.

bus_i	bus_type	Pd	Qd	..	Vm	Va	base_kV	zone	Vmax	Vmin
1	3	0	0	…	1	0	132	1	1.05	0.95
2	1	0	0	…	0.9995	-0.0812	132	1	1.05	0.95
..	..	..	..	…	..	..	..	..	..	..
17	1	0	0	…	0.9677	-8.8311	66	1	1.05	0.95

The data columns in Table 3 are identical to the columns in the MATPOWER bus format:

• bus_i: Bus (node) number (positive integer)
• bus_type: Bus type (1 = PQ, 2 = PV, 3 = ref, 4 = isolated). Buses in the grid with at least one dispatchable/controllable generator are modeled as a PV bus (type 2).
• Pd: Real power for peak load demand (MW)
• Qd: Reactive power for peak load demand (MVAr)
• Gs: Shunt conductance (MW demanded at *V* = 1.0 p.u.)
• Bs: Shunt susceptance (MVAr injected at *V* = 1.0 p.u.)
• bus_area: Area number (positive integer)
• Vm: Voltage magnitude (p.u.)
• Va: Voltage angle (degrees)
• base_kV: Base voltage (kV)
• zone: Loss zone (positive integer)
• Vmax: Maximum voltage magnitude (p.u.)
• Vmin: Minimum voltage magnitude (p.u.)

The complex voltage values stored in the Vm and Va columns are the solution of an AC power flow calculation for the reference case (see Table 2), using the load and generation values for this point in time.

The values in the Vmax and Vmin specify bus voltage limits ± 0.05 p.u. for the grid, which are voltage limits that are commonly used as planning criteria. Power flow was also solved for every hour of the 2019-2022 load and generation time series, converging in all cases with no violations of the declared voltage limits or line/transformer ratings in Table 5. Fig. 2 shows the bus voltage duration curve for all 17 buses, based on hourly AC power flow for the intact grid for the full 2019-2022 time series.

**Fig. 2:**
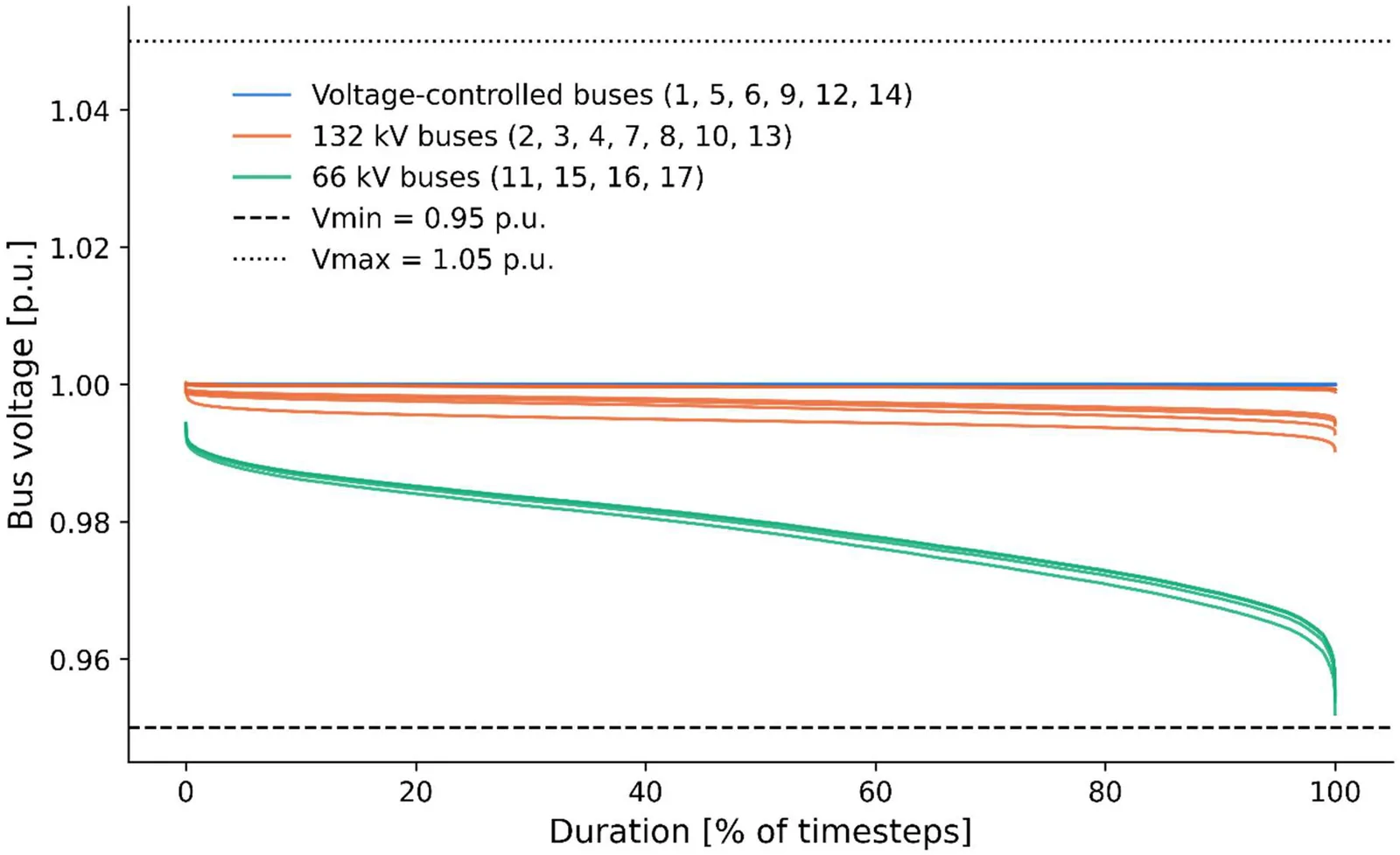
Bus voltage duration curves for the intact grid. Based on AC power flow results for the full 2019-2022 hourly time series.

Table 4 shows the data file SecurEL_reference_grid_base_bus_extra.csv. It is a mapping of which buses have which loads and generators. It is not standard MATPOWER format, but it is useful information for mapping load and generation time series to the correct buses.

**Table 4:** Extra bus data from SecurEL_reference_grid_base_bus_extra.csv.

bus_i	Loads	Generators
1		
2		
3	L1, L2	
4		
5	L3	G1, G2
6	L4	G3, G4, G5, G6
7	L5, L6	
8	L7	
9	L8, L9	G7, G8, G9
10	L10	
11	L11	
12	L12	G10, G11, G12
13	L13	G13, G14
14	L14	G15, G16, G17
15	L15	
16	L16	
17		

Table 5 shows an excerpt of the contents in the data file SecurEL_reference_grid_base_branch.csv.

**Table 5:** Example of branch data from SecurEL_reference_grid_base_branch.csv.

f_bus	t_bus	br_r	br_x	br_b	rate_A	rate_B	rate_C	tap	shift	br_status
1	2	1.18E-05	8.16E-5	1.00E-05	320.07	320.07	320.07	0	0	1
2	3	6.08E-4	2.03E-3	1.00E-05	167.58	167.58	167.58	0	0	1
…	…	…	…	…	…	…	…	…	…	…
15	17	5.34E-4	8.12E-4	1.00E-05	74.30	74.30	74.30	0	0	1

The data columns in Table 5 are identical to the columns in the MATPOWER branch format:

• f_bus: “From” bus (node) number
• t_bus: “To” bus (node) number
• br_r: Resistance (p.u.)
• br_x: Reactance (p.u.)
• br_b: Total line charging susceptance (p.u.)
• rate_A: MVA rating A (long term rating), set to 0 for unlimited (or unknown)
• rate_B: MVA rating B (short term rating), set to 0 for unlimited (or unknown)
• rate_C: MVA rating C (emergency term rating), set to 0 for unlimited (or unknown)
• tap: Transformer off nominal turns ratio, (taps at “from” bus, impedance at “to” bus, i.e., if r = x = b = 0, tap = |Vf|/|Vt|, where Vf and Vt are bus voltages at the from and to bus, respectively)
• shift: Transformer phase shift angle (degrees), positive ⇒ delay
• br_status: Initial branch status, 1 = in-service, 0 = out-of-service

A note on the line ratings is that rate_B and rate_C was set equal to rate_A since this information was not available.

The solution of an AC power flow calculation for the reference case in Table 2 for the branches is stored in the file SecurEL_reference_grid_base_branch_pf_sol.csv, with one row per branch matching SecurEL_reference_grid_base_branch.csv, and the following columns:

• p_from_mw: Active power flow into the branch at the ``from'' bus (MW)
• q_from_mvar: Reactive power flow into the branch at the ``from'' bus (MVAr)
• p_to_mw: Active power flow into the branch at the ``to'' bus (MW)
• q_to_mvar: Reactive power flow into the branch at the ``to'' bus (MVAr)

Table 6 shows an excerpt of the data file SecurEL_reference_grid_base_branch_extra.csv that defines data on the branches in addition to those data included in the MATPOWER branch format.

**Table 6:** Example of additional branch data from SecurEL_reference_grid_base_branch_extra.csv

f_bus	t_bus	l_km	type	Voltage_kV	asset_type	installation_year	location_type
1	2	0.5	525-AL1/68-ST1A	132	OH_line	2003	rural
2	3	10	239-AL1/39-ST1A	132	OH_line	2011	rural
..	..	..	..	..	..	..	..
15	17	1.5	TSLE 3 × 1 × 500 Al	66	Cable	1988	urban

The data columns in SecurEL_reference_grid_base_branch_extra.csv are defined as follows:

• f_bus: “From” bus number
• t_bus: “To” bus number
• l_km: Line length (kilometres); blank for the branch that is a transformertype: Line type according to the Norwegian type designation [7]
• Voltage_kV: The voltage (in kV) that the branch (line or transformer) is operated at.
• asset_type: The type of asset: either OH_line (overhead line), Cable (underground cable) or trafo (transformer).
• installation_year: The year the asset was installed
• location_type: The type of location of the line (urban, semi-urban or rural), classification from [7]

Table 7 shows an excerpt of the data file SecurEL_reference_grid_base_gen.csv. There is one row per generator in the grid, 17 rows in total.

**Table 7:** Example of generator data from SecurEL_reference_grid_base_gen.csv.

bus	Pg	Qg	Qmax	Qmin	Vg	mBase	status	Pmax	Pmin
5	10.0	0.0	4.8	-4.8	1.0	10.0	1	10.0	0.0
5	0.0	0.0	0.384	-0.384	1.0	10.0	1	0.8	0.0
..	..	..	..	..	..	..	..	..	..
14	0.0	0.0	5.616	-5.616	1.0	10.0	1	11.7	0.0

The data columns in Table 7 are identical to the columns in the MATPOWER «gen» format:

• bus: Bus number
• Pg: Real power output (MW) for the reference case (the system peak net-load hour)
• Qg: Reactive power output (MVAr), set to 0 for all generators
• Qmax, maximum reactive power output (MVAr) given as 0.48*Pmax
• Qmin, minimum reactive power output (MVAr) given as -0.48*Pmax
• Vg, voltage magnitude setpoint (p.u.) set to 1 for all
• mBase, set to the default baseMVA = 10 for all
• Status: 1 (machine in service), 0 (machine out of service)
• Pmax: Maximum real power output (MW), equals the installed power
• Pmin: Minimum real power output (MW), set to 0 for all

Table 8 shows an excerpt of the data file SecurEL_reference_grid_base_gen_extra.csv. It gives information about all of the generators in the grid. The “control_mode” variable is either “dispatchable” or “fixed_profile”. All generators above 1.5 MW are “dispatchable”. This is according to the national guidelines for functional requirements in the power system [8] which requires all power plants over 1.5 MW to participate in voltage regulation. Yearly average generation is given in GWh.

**Table 8:** Example of additional generator data from SecurEL_reference_grid_base_gen_extra.csv.

generator_name	control_mode	p_max_mw	yearly_avg_generation_gwh
G1	dispatchable	10	50.3
G2	fixed_profile	0.8	3.23
..	..	..	..
G17	dispatchable	11.7	31.8

When modelling grid reinforcement or expansion alternatives in grid planning studies, component-level technical parameters are required. Table 9, Table 10 contains this data for standard overhead line and cable types, from [7].

**Table 9:** The contents of the data file standard_overhead_line_types.csv. Source: [7]

Type	R_ohm_per_km	X_ohm_per_km	Cd_nF_per_km	Imax_A
112-AL1/18-ST1A	0.260	0.403	9.067	518
151-AL1/25-ST1A	0.193	0.419	8.756	629
191-AL1/31-ST1A	0.153	0.411	8.926	733
239-AL1/39-ST1A	0.123	0.404	9.090	846
293-AL1/48-ST1A	0.101	0.398	9.243	966
381-AL1/62-ST1A	0.078	0.390	9.453	1144
402-AL1/52-ST1A	0.074	0.390	9.470	1178
476-AL1/78-ST1A	0.063	0.383	9.634	1321
525-AL1/68-ST1A	0.057	0.381	9.694	1400

**Table 10:** The contents of the data file standard_cable_types.csv. Source: [7]

Type	R_ohm_per_km	X_ohm_per_km	Cd_uF_per_km	Imax_A
TSLE 3 × 1 × 150 Al / 25	0.206	0.20	0.14	370
TSLE 3 × 1 × 240 Al / 35	0.125	0.18	0.16	465
TSLE 3 × 1 × 300 Al / 35	0.100	0.18	0.17	500
TSLE 3 × 1 × 400 Al / 35	0.0778	0.17	0.19	590
TSLE 3 × 1 × 500 Al / 50	0.0605	0.16	0.21	650
TSLE 3 × 1 × 630 Al / 50	0.0469	0.15	0.25	715
TSLE 3 × 1 × 800 Al / -	0.0367	0.15	0.28	780
TSLE 3 × 1 × 1000 Al / -	0.0291	0.14	0.31	850
TSLE 3 × 1 × 1200 Al / -	0.0247	0.14	0.33	900
TSLE 3 × 1 × 1600 Al / -	0.0186	0.13	0.37	990
TSLE 3 × 1 × 2000 Al / -	0.0149	0.13	0.41	1050

Unit costs are not included. The current edition of the Handbook for grid planning [7] does not provide updated cost figures for these standard types, and no other source with comparable cost data across all listed types was identified. Cost-based network planning studies using this dataset will need to supply their own component cost assumptions.

The data column titles in Table 9, Table 10 are defined as follows:

• type: Line type according to [7]
• R_ohm_per_km: Resistance in Ω per km
• X_ohm_per_km: Reactance in Ω per km
• Cd_nF_per_km/ Cd_uF_per_km: Mutual capacitance Cd of the lines in nF or µF per km
• Imax_A: Current carrying capacity Imax in ampere represents the maximally allowed continuous current of the lines/cables, at the standard ambient temperature of 20 °C.

The data source for the standard line data is [7].

### 3.2 Load data

The load data associated with the reference grid consists of a set of data files with hourly load values from 1^st^ of January 2019 at 01:00 to the 31^st^ of December 2022 at 23:00, four years in total. Each load represents one MV feeder. The feeders go mainly to consumers, with some small run-of-river hydropower as well. An overview of the load data files, including load prognoses, are given in Table 11.

**Table 11:** Overview of data files in the load dataset.

File name	Description of data
loads_timeseries.csv	Set of 16 hourly load time series for 2019-2022
SecurEL_reference_grid_prognosis_base.csv	A base (“most likely”) prognosis for how the peak net load of the bus/node will evolve over the next 20 years
SecurEL_reference_grid_prognosis_high.csv	A high (“optimistic”) prognosis for how the peak net load of the bus/node will evolve over the next 20 years

In Table 12, an excerpt from the load timeseries file is shown. The time series contains no duplicate timestamps and is continuous over the full 2019-2022 period, except for a single 24-hour gap on 2020-12-31.

**Table 12:** Example of the load time series from loads_timeseries.csv, showing the first four and the last timesteps.

Timestamp	L1 (MWh)	L2 (MWh)	…	L15 (MWh)	L16 (MWh)
01.01.2019 01:00	3.69	121.34	…	15.13	12.88
01.01.2019 02:00	3.14	127.8	…	16.18	12.4
01.01.2019 03:00	2.75	121.93	…	16.31	12.61
01.01.2019 04:00	2.9	121.25	…	16.61	12.37
…	…	…	…	…	…
31.12.2022 23:00	2.52	121.4	…	16.92	15.21

Table 13 is included to give an overview of all of the load timeseries. Min (MW) is the minimum value, Avg (MW) is the average value, Max (MW) is the maximum value, Std (MW) is the standard deviation and P95 (MW) is the value which 95 % of the recorded loads fall below. Some of the loads’ minimum values are negative. This is because some of the feeders have run-of-river hydropower production connected to them, and in some of the recorded hours, the feeder is net *exporting* power, not consuming it. The main customer type is also included. Main customer type was determined by expert judgement by the data owner, based on which customer type dominates each load/feeder. The individual loads may supply a mixture of customer categories.

**Table 13:** Statistical information about the load data. Min, Avg, Max, Std, and P95 denote the minimum, average, maximum, standard deviation, and 95th percentile of the time series, respectively.

Load ID	Main customer type	Min (MW)	Avg (MW)	Max (MW)	Std (MW)	P95 (MW)
L1	Residential	-3.92	2.64	11.07	2.17	6.23
L2	Industry	10.91	106.24	188.17	21.71	143.01
L3	Residential	1.88	8.78	16.49	2.69	13.17
L4	Residential	-0.70	1.23	2.99	0.55	2.15
L5	Residential	1.81	10.96	23.15	3.81	17.32
L6	Residential	3.73	15.61	31.12	4.77	24.00
L7	Residential	2.92	13.27	27.72	4.45	20.71
L8	Residential	0.07	2.32	5.37	0.87	3.73
L9	Residential	-0.95	1.12	3.64	0.67	2.25
L10	Residential	3.15	15.56	33.38	5.33	24.55
L11	Residential	0.69	7.50	15.86	2.57	11.96
L12	Residential	2.40	7.32	13.34	1.70	10.10
L13	Residential	-0.20	1.43	3.43	0.50	2.30
L14	Residential	1.40	9.06	17.62	2.89	13.85
L15	Industry	3.89	14.08	31.50	4.11	21.34
L16	Commercial	2.08	11.96	23.83	3.70	18.18

Table 14 shows a base (``most likely'') peak net load prognosis per bus/node in the grid in a 20-year perspective (2027-2047). The values are normalized to the current maximal net load value of the station. The high (“optimistic”) peak net load prognosis table is also included in the dataset and is on the same format. The empty rows in Table 12 represent buses without any net load. Note that the prognoses give the *net* peak load factor for the bus/node, not just for the demand: Some buses have both load and production, and the prognosis factor is for scaling the sum of load minus production – hence the term *net* load prognosis.

**Table 14:** Example of peak net load prognosis data from SecurEL_reference_grid_prognosis_base.csv.

bus_i	2027	2028	…	2047
1			…	
2			…	
3	1.00	1.00	…	1.16
4			…	
5	1.01	1.02	…	1.48
…	…	…	…	…
17			…	

The intended use of the peak net load prognosis factors is to scale net load (load demand minus generation) for grid planning studies which typically analyse the peak net load growth in a 20-year perspective.

### 3.3 Generation data

Table 15 shows an overview of the generation data contained in the dataset. See also Chapter 4.1 where the files SecurEL_reference_grid_base_gen.csv and SecurEL_reference_grid_base_gen_extra.csv give more information about the generators.

**Table 15:** Overview of data files in the hydropower generation dataset.

File name	Description of data
generation_timeseries.csv	Set of hourly synthetic generation time series for 2019-2022

In Table 16, an excerpt from the generation timeseries file is shown. The time series contains no duplicate timestamps and is continuous over the full 2019-2022 period, except for one 24-hour gap on 2020-12-31.

**Table 16:** Example of the generation time series from generation_timeseries.csv, showing the first four and the last timesteps.

Timestamp	G1 (MWh)	G2 (MWh)	…	G16 (MWh)	G17 (MWh)
01.01.2019 01:00	0.0	0.0	…	1.93	0.0
01.01.2019 02:00	0.0	0.0	…	1.92	0.0
01.01.2019 03:00	0.0	0.0	…	1.92	0.0
01.01.2019 04:00	0.0	0.8	…	1.92	0.0
…	…	…	…	…	…
31.12.2022 23:00	0.0	0.0		1.118133	11.7

Table 17 is, as for the load timeseries, included to give an overview of all the generator data.

**Table 17:** Statistical information about the generation data. Min, Avg, Max, Std, and P95 denote the minimum, average, maximum, standard deviation, and 95th percentile of the time series, respectively.

Generator ID	Min (MW)	Avg (MW)	Max (MW)	Std (MW)	P95 (MW)
G1	0.0	5.75	10.0	4.9	10.0
G2	0.0	0.37	0.8	0.4	0.8
G3	0.0	15.72	30.0	14.63	30.0
G4	0.0	26.52	48.0	21.93	48.0
G5	0.0	47.63	118.0	49.42	118.0
G6	0.0	36.94	66.0	30.69	66.0
G7	0.0	8.76	15.0	7.3	15.0
G8	0.0	0.54	1.8	0.82	1.8
G9	0.0	0.04	0.1	0.05	0.1
G10	0.0	2.52	8.6	3.89	8.6
G11	0.0	1.35	2.0	0.64	2.0
G12	0.0	18.49	32.0	15.47	32.0
G13	0.0	0.68	1.0	0.32	1.0
G14	0.0	0.34	0.5	0.16	0.5
G15	-0.0	2.32	3.3	1.1	3.3
G16	-0.0	2.62	3.7	1.22	3.7
G17	0.0	3.63	11.7	5.35	11.7

Fig. 3 shows a one-week section of the load and generation time series, together with the resulting net import at the EHV/HV connection (bus 1), centred on the documented system peak net-load hour.

**Fig. 3:**
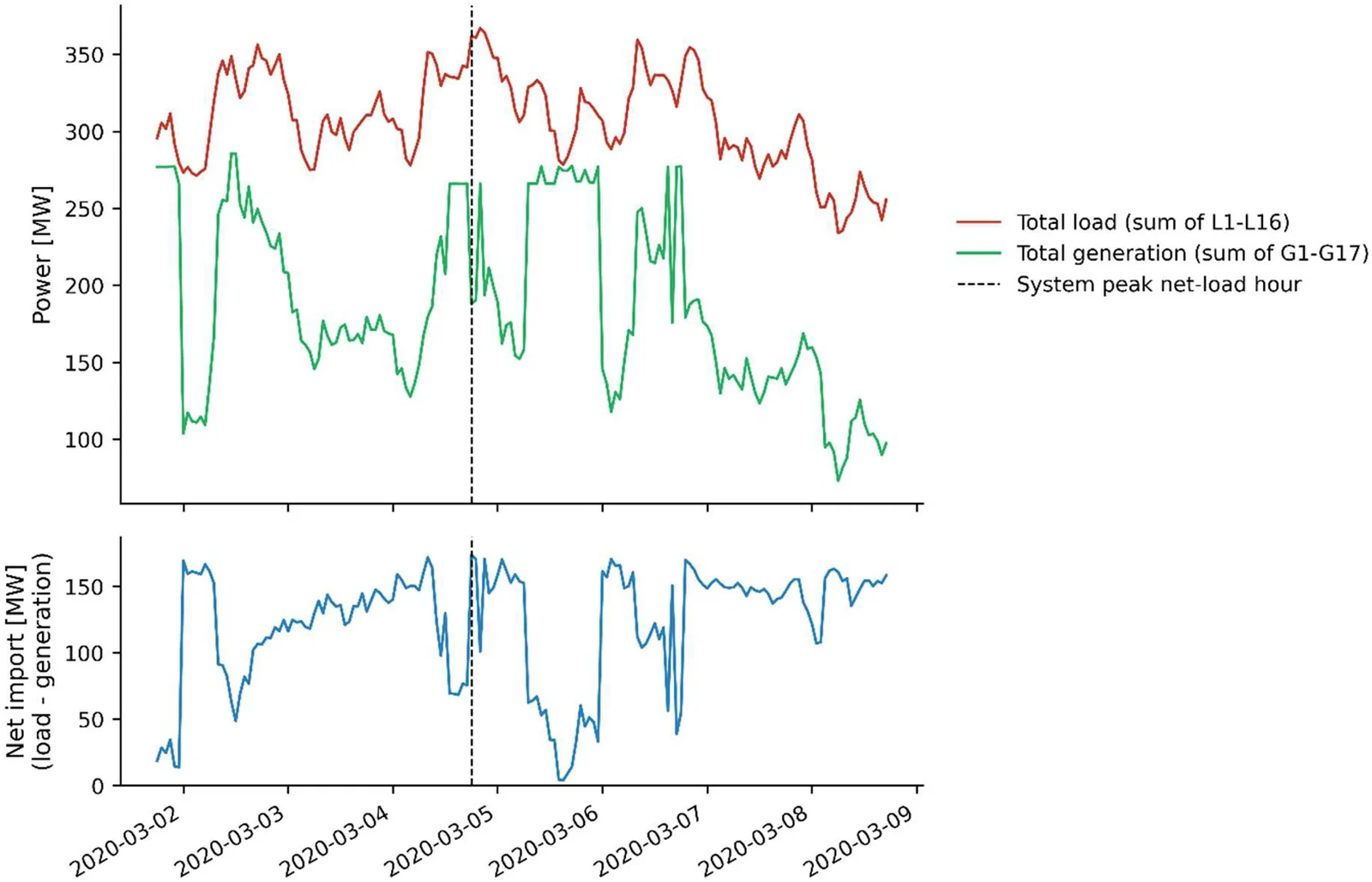
One-week section of the SecurEL reference system time series, centred on the system peak net load hour (2020-03-04 18:00).

### 3.4 Power transfer corridors

The SecurEL reference system has four power transfer corridors that are under pressure from increasing demand. Historical bottlenecks, selected by expert judgement by the data owner as the cut-sets used in the DSO's own network-planning assessments, are presented in Table 18. The corridor capacity is the capacity of the limiting line (Line 1, with the lowest capacity). It is often called the “N-1 capacity”. The corridor’s loading is the limiting line’s loading in case of a contingency (outage of Line 2), computed by running a power flow for every hour with the other line taken out of service. This subchapter presents duration curves for the limiting line in each corridor to give the reader an understanding of the current degree of loading in the SecurEL reference system. The dotted ``100 % line loading'' limit shown refers to the N-1 capacity.

**Table 18:** An overview of the power transfer corridors under pressure and the power lines that comprise them

Power transfer corridor #	Line 1 (Limiting line)	Line 2 (Critical contingency)
1	Bus 3→ Bus 5	Bus 7→Bus 10
2	Bus 3→ Bus 5	Bus 6→Bus 10
3	Bus 2→Bus 3	Bus 2→Bus 3
4	Bus 3→Bus 6	Bus 6→Bus 10

Fig. 4, Fig. 5, Fig. 6 shows that although the N-1 criterion is a typical planning criterion for HV distribution grids, the current state of the grid surpasses the limit for all but one power transfer corridor, at least for a few hours per year.

**Fig. 4:**
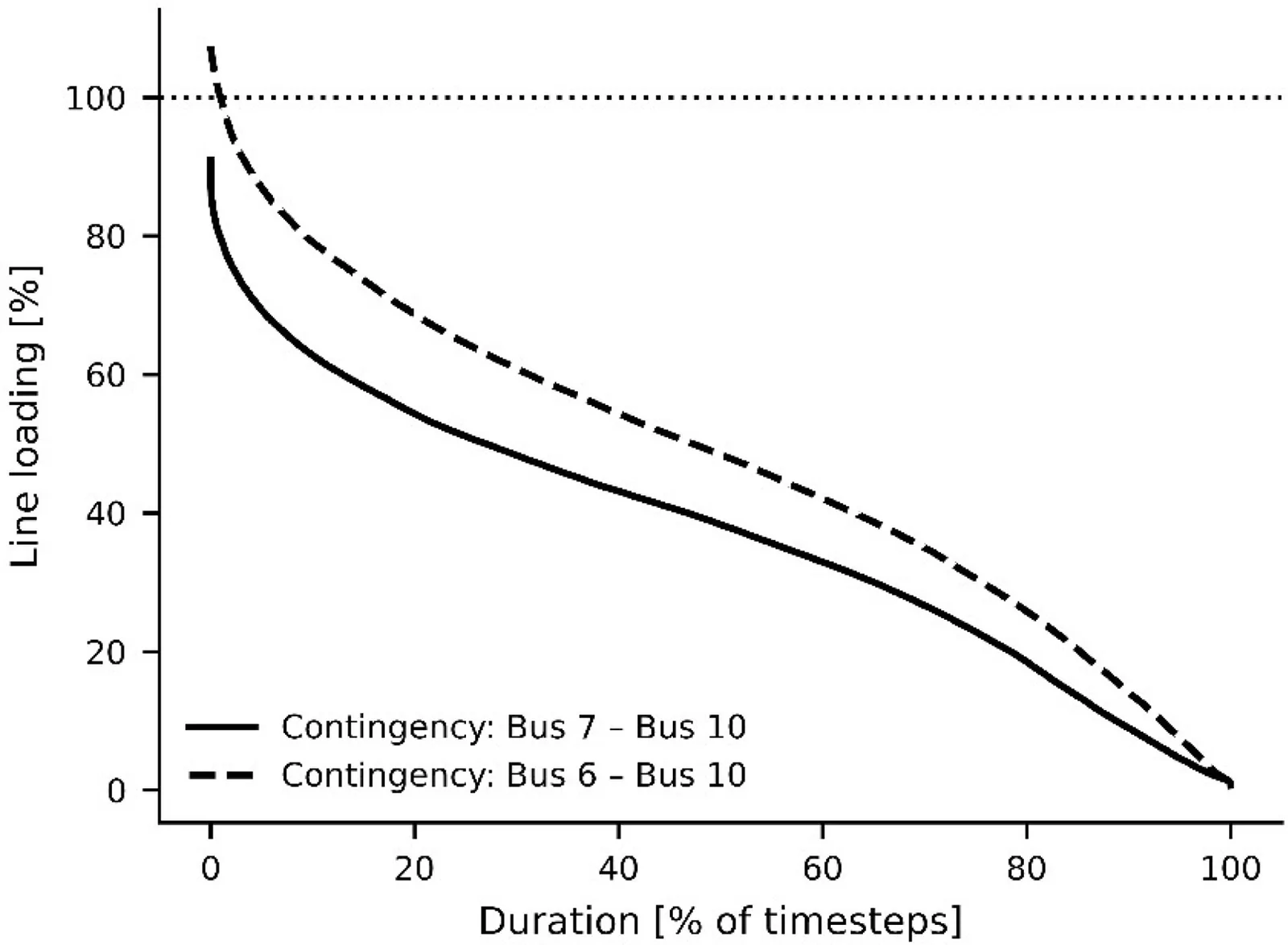
Duration curves for power transfer corridors 1 and 2, which are the duration curves for line from Bus 3→Bus 5 with contingencies of lines from Bus 7→ Bus 10 and from Bus 6→ Bus 10, respectively.

**Fig. 5:**
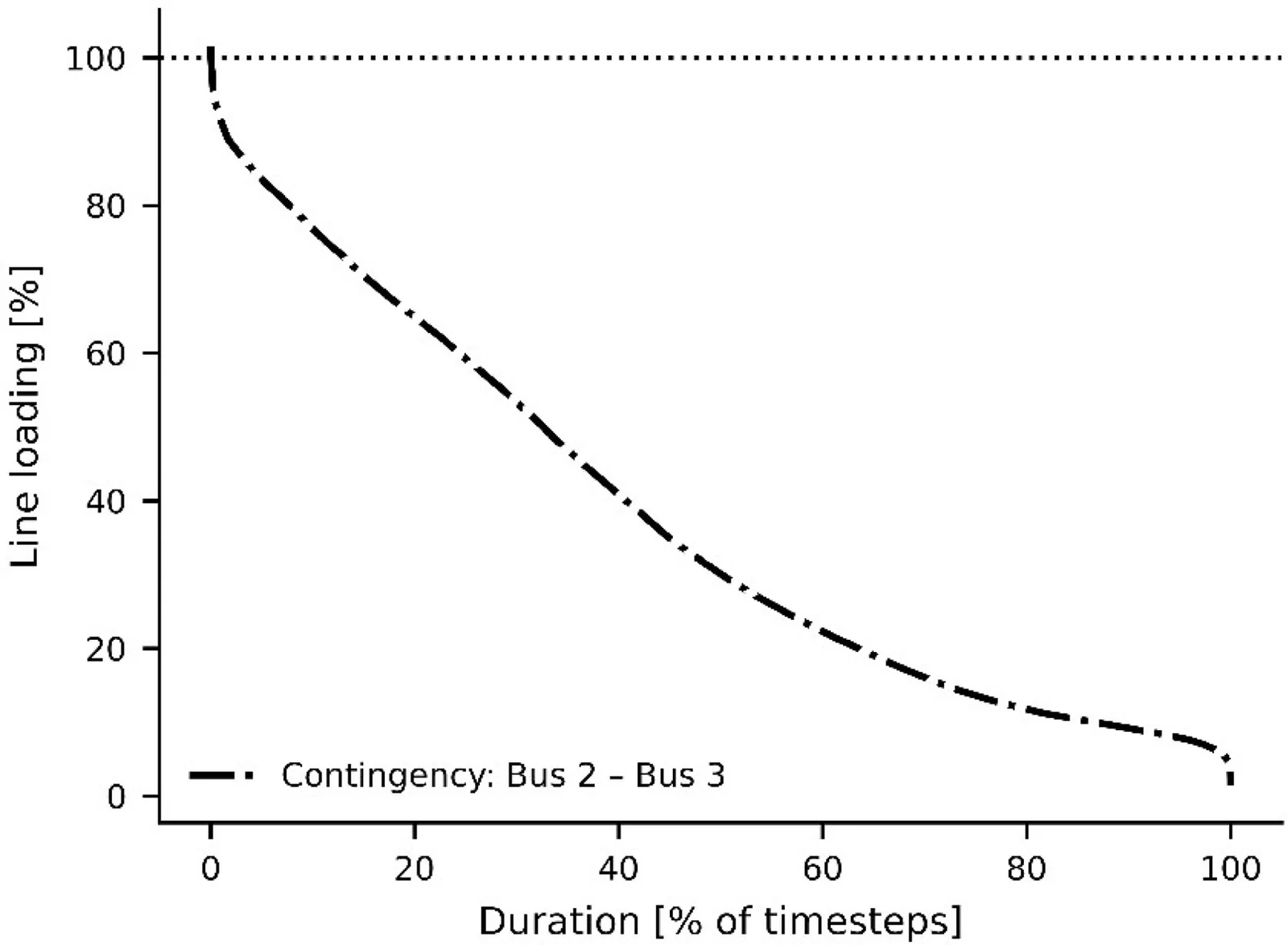
Duration curve for power transfer corridor 3, which is the duration line for line from Bus 2→ Bus 3 with a contingency on its parallel, identical line.

**Fig. 6:**
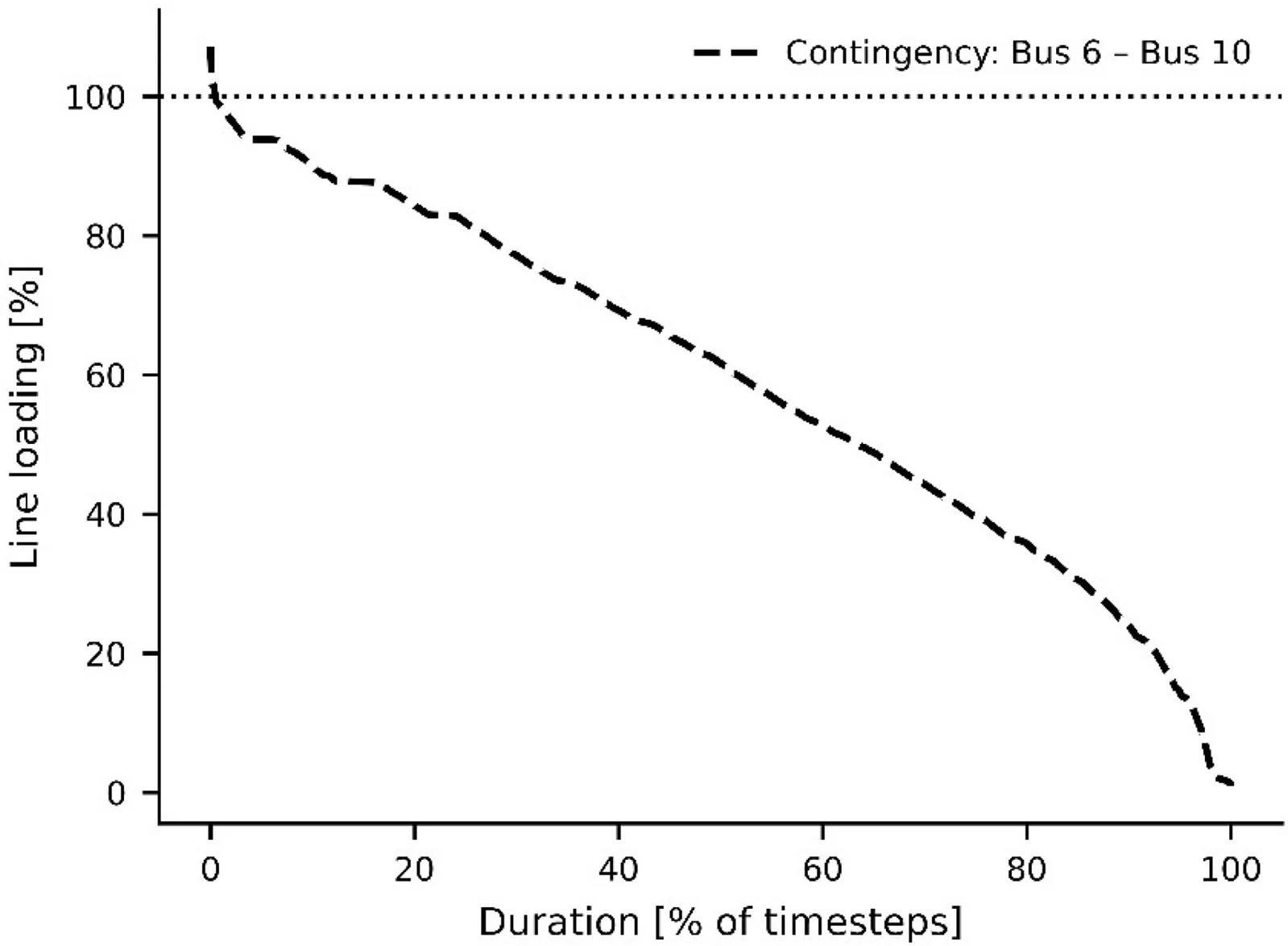
Duration curve for power transfer corridor 4, which is the duration curve for line from Bus 3→Bus 6 with contingency of line from Bus 6→Bus 10.

### 3.5 Additional information

Table 19 contains an overview of data files with additional information about the SecurEL reference system.

**Table 19:** Overview of data files containing additional information.

File name	Description of data
oh_lines_aar_50.csv	Ambient-adjusted line ratings (AAR) for overhead lines in the grid dataset, given a maximum allowable conductor temperature of 50 °C
oh_lines_aar_80.csv	AAR for overhead lines in the grid dataset, given a maximum allowable conductor temperature of 80 °C
reldata_for_component_types.csv	Reliability statistics for types of components in the grid dataset
temperature_time_series.csv	Temperature time series from Stavanger (Western Norway)

Table 20 shows the contents of the file oh_lines_aar_80.csv, which shows the maximum allowed current (in A) for different values of the ambient temperature, from -30 °C to +30 °C given a maximum allowable conductor temperature of 80 °C. A similar table is found in oh_lines_aar_50.csv, for a maximum allowable conductor temperature of 50 °C. These ratings represent steady-state ampacity limits for each fixed ambient condition.

**Table 20:** The contents of the file oh_lines_aar_80.csv, ambient-adjusted ratings (AAR) given various ambient temperatures.

Conductor type	-30 °C [A]	-20 °C [A]	-10 °C [A]	0 °C [A]	10 °C [A]	20 °C [A]	30 °C [A]
525-AL1/68-ST1A	1681	1607	1527	1441	1347	1244	1128
239-AL1/39-ST1A	1028	982	933	880	823	760	691
151-AL1/25-ST1A	769	735	698	658	616	569	517
191-AL1/31-ST1A	893	853	811	764	715	661	600
381-AL1/62-ST1A	1378	1317	1252	1181	1104	1020	926
293-AL1/48-ST1A	1167	1115	1059	999	934	863	783
112-AL1/18-ST1A	636	608	577	544	509	470	428

Table 21 shows the contents of the data file reldata_for_component_types.csv, which shows average failure rates per system component derived from the FASIT system [9], based on reported failure data for all of Norway for the years 2015-2025 [10]. The raw disturbance and outage data underlying these statistical data are confidential. The column titles are explained below:

• lambda_perm: Failure frequency for permanent faults (per year per 100 km conductor (for overhead lines and underground tables), or per year per 100 components for transformer)
• lambda_temp: Failure frequency for temporary faults (per year per 100 km conductor (for overhead lines and underground tables), or per year per 100 components for transformer)

**Table 21:** The contents of the file reldata_for_component_types.csv.

main_type	lambda_perm	lambda_temp
Overhead line (110-132 kV)	0.24	0.59
Underground cable (33-66 kV)	0.58	0.06
Transformer	0.49	0.31

``Temporary fault'' denotes an automatically resolved fault, and ``permanent fault'' a fault requiring repair before reconnection [9].

Table 22 shows temperature time series data from Stavanger, a city in Western Norway. This data is relevant due to the load data’s dependence on ambient temperature and also for calculating the AAR. The data is weather station data as provided by the Norwegian Meteorological Institute [1]. The temperature column values are given in degrees Celsius. The time series contains no duplicate timestamps, and two gaps (2-3 September 2019, 17 h; 11-12 July 2021, 24 h) inherited from the weather station’s record.

**Table 22:** Example of the temperature time series from temperature_time_series.csv. Source: [1]

Name	Station	Timestamp	Air_temperature_C
Stavanger - Våland	SN44640	01.01.2019 01:00	7.10
Stavanger - Våland	SN44640	01.01.2019 02:00	6.20
…	…	…	…
Stavanger - Våland	SN44640	31.12.2022 23:00	1.00

## 4 Experimental Design, Materials and Methods

The raw dataset that the SecurEL reference system dataset is based on included detailed power system information from the distribution system operator’s network information system as well as measurements from regional distribution station feeders, which was hourly timeseries data from 2019-2022 for both load and generation. In Norway, both load data, generation data and grid asset data can be classified as sensitive power system information, especially when they are combined. Publishing such data *as-is* could increase the risk of identifying critical infrastructure characteristics or commercially sensitive data about larger power-intensive consumers.

Hence, the dataset was processed to enable open dissemination while protecting sensitive power system information. In the following subchapters, the data processing is described in detail: In 5.1, the method for gathering the grid dataset; in 5.2, the load data anonymization procedure; in 5.3, the model for generating synthetic production data and in 5.4 the method and assumptions for generating ambient-adjusted rating (AAR) data. Finally, subchapter 5.5 describes how the power system reliability data was gathered, and subchapter 5.6 summarizes the provenance of every major dataset component.

### 4.1 Grid data

The grid topology in this dataset is retained from the original raw grid dataset obtained from the distribution system operator's network information system, since the topology graph alone is not considered identifying information. Branch types are substituted with standard types from the Handbook for grid planning [7], and line lengths are estimated using the public NVE Atlas database [11] to obtain realistic geographic distances without disclosing the network's real location. The branch electrical parameters themselves are retained from the original grid dataset. The dataset remains a simplification of the original grid in other respects, since components such as switches are not included. For the transformer data used in the dataset, a standard type was used: 63 MVA 110/20 kV as defined in pandapower’s standard types [12], which is based on DIgSILENT PowerFactory [13]. As can be seen in Fig. 1, there are two trafos of this type in parallel.

### 4.2 Load data

The raw dataset consisted of hourly electricity consumption time series for each bus/node in the grid. The raw dataset contains data which may reveal potentially sensitive power system information, such as a temperature dependency that could reveal where in Norway the grid is located. Therefore, a load anonymization procedure was performed, and is described in this subchapter. The anonymization procedure consists of three steps: temperature separation, spectral load modelling and relocation to a new climate.

### 4.2.1 Temperature separation

Each hourly load value in the raw time series is decomposed into a temperature dependent component and a temperature-independent base load using a linear load-temperature dependency based on the FASIT model [9]. The model estimates hourly average temperature sensitivity based on the average temperature in the last 24 h. It accounts for the space heating threshold (T = 15 °C) over which barely any electricity is used for space heating, as well as separating between working days, weekends and public holidays. If L(t) is the raw load time series and T(t) the ambient local temperature, the FASIT load model estimates a temperature dependency f(T(t)) which can be subtracted from the raw load time series, leaving a resulting base load B(t):

(1) B(t)=L(t)−f(T(t))

The base load B(t) is the load time series with the influence of the local weather removed.

### 4.2.2 Spectral load modeling

The temperature-independent base load B(t) is decomposed into a periodic component and a stochastic residual component. This step aims to capture the dominant seasonal and daily demand patterns while modeling the remaining variability or noise using a stochastic process.

A periodic component is estimated using fast Fourier transform (FFT) and periodic boundary conditions, which makes a smooth yearly profile. The remaining signal is organized into daily load profiles in the same way as for the yearly profile. It separates the data into workdays and weekends. Finally, the periodic signal is constructed by adding the daily profile to the yearly one.

(2) $$P\left(t\right)=P_{day}\left(t\right)+P_{year}\left(t\right)$$

The residual component R(t) is the difference between the baseline and the periodic signal:

(3) R(t)=B(t)−P(t)

The residual is modelled as an Ornstein-Uhlenbeck (OU) stochastic process [14] which is a mean-reverting continuous-time process commonly used to represent autocorrelated noise. The process has a time-varying diffusion parameter which preserves the temporal variability of the original residuals, which generally are larger when the demand is large. Generating a random realization of this process ${\tilde{R}}_{OU}\left(t\right)$, will have similar variance and autocorrelation structure as the original. The periodic signal and the OU noise realization can be combined to a synthetic base load $\tilde{B}\left(t\right):$

(4) $$\tilde{B}\left(t\right)=P\left(t\right)+\;{\tilde{R}}_{OU}\left(t\right)$$

### 4.2.3 Relocation to new climatic conditions

In the final step, the synthetic base load $\tilde{B}\left(t\right)$ is re-combined with the temperature-dependent function f(T(t)) found in the first step, using a temperature time series from a new geographical location, $T_{new}\left(t\right)$. The final anonymized time series, $\tilde{L}\left(t\right)$, is given by

(5) $$\tilde{L}\left(t\right)=\tilde{B}\left(t\right)+\;f\left(T_{new}\left(t\right)\right)$$

$T_{new}\left(t\right)$ is included in the dataset, see Table 22 in subchapter 4.5.

### 4.2.4 Validation of the anonymization procedure

To quantify how the anonymization procedure described above affects the statistical properties of the load data, the anonymized time series were compared against the original (pre-anonymization) time series. Four normalized/relative measures were computed: (1) peak load factor (mean load divided by peak load), reported as the ratio between the anonymized and original series; (2) ramp rate, the mean absolute hour-to-hour change normalized by the mean load, also reported as a ratio; (3) autocorrelation at lags of 1 h, 24 h and 168 h (a week), reported as the absolute deviation between the anonymized and original series; and (4) inter-load correlation, the pairwise Pearson correlation between loads, compared before and after anonymization.

These measures are reported in aggregated form: mean, minimum and maximum across the 16 loads, see Table 23, Table 24.

**Table 23:** Aggregated statistics comparing the anonymized and original load time series (16 loads, L1-L16).

Measure	Mean	Min	Max
Peak load factor (anonymized/original ratio)	1.041	0.794	1.309
Normalized ramp rate (anonymized/original ratio)	1.859	1.406	2.429
Autocorrelation 1h, abs. deviation	0.007	0.003	0.009
Autocorrelation 24h, abs. deviation	0.139	0.083	0.442
Autocorrelation 168h (week), abs. deviation	0.209	0.069	0.594

**Table 24:** Inter-load correlation, before and after anonymization (120 site pairs among 16 loads).

N pairs	Mean abs. deviation	Max abs. deviation	Correlation-of-correlations
120	0.073	0.221	0.987

Short-term temporal structure is well preserved: the 1-hour autocorrelation deviates on average by only 0.007 (maximum 0.009) between the original and anonymized series, across all 16 loads. Autocorrelation at daily and weekly scale degrades more for a subset of loads: at 24 h the mean deviation is 0.14 (maximum 0.44), and at 168 h (one week) the mean deviation is 0.21 (maximum 0.59). The weekly cycle is therefore the least robust of the four measures to the anonymization method for the most affected load sites.

The peak load factor is, on average, unchanged (mean ratio 1.04 across the 16 loads), but individual series vary in both directions (0.79-1.31); there is thus no systematic bias, though the spread across individual series is not negligible.

The ramp rate is elevated in the anonymized series for all 16 loads (ratio range 1.41-2.43, mean 1.86). Because the deviation points in the same direction for every load, this reflects a systematic effect in the anonymization method, likely an increase in high-frequency variance, and is treated as a known limitation of the dataset (see Limitations section).

The inter-load correlation structure is well preserved at the aggregate level: the correlation-of-correlations between the two 16 × 16 correlation matrices is 0.99, and the mean absolute deviation per site pair is only 0.07. The worst individual pair deviates by 0.22, so the method preserves acceptable, but not exact, pairwise relationships for all site combinations.

A methodological caveat applies to all measures above: the anonymized series is also moved to a new location with a different temperature profile than the original location. The deviations reported here therefore reflect the combined effect of the anonymization method and the relocation.

### 4.3 Generation data

Power generation time series were obtained through simulations with the PowerGAMA tool [15]. This is a lightweight power market simulator available on GitHub [16] that iterates over timesteps and finds the cost-based optimal dispatch in each timestep.

In PowerGAMA, the power system is represented as a set of generators and loads and their connection to the power grid. The grid and load datasets described in this article were used as the input. The power balance (import/export) at the main feeder was also provided as input and modelled as a fixed load with positive or negative values. All the generators were assumed to be hydropower plants with storage in the form of water reservoirs. Reservoir sizes were set according to what are typical values in Norway, as found using publicly available information from the NVE hydropower database^1^: For generators larger than 10 MW, storage capacities were assumed to be 2300 h times generator capacity, and for smaller generators, they were assumed to be 400 h times generator capacities. Water inflow was assumed to follow the same pattern as Norway as a whole for the given time period, the same for all generators. Marginal costs of the generators were set according to reservoir filling levels, using a generic water value curve provided as input data. This curve has high values when the filling level is low and goes to zero when the filling approaches 100%. The reservoir, inflow, and water-value-curve assumptions used as PowerGAMA inputs are not included in the released dataset, since they reveal specific power-system information beyond what the anonymized load and generation time series already disclose. Consequently, the full generation-dispatch modelling process cannot be reproduced from this dataset.

At each time step, the power generation is determined by solving a linear optimization problem that minimizes the total system generation cost while satisfying demand and respecting generation and grid constraints (linearized optimal power flow). The resulting output time series are consistent with grid constraints, system demand and hydropower availability/reservoir behavior.

In order to ensure that the generation data satisfied the same security requirements as the original dataset, a preventive security-constrained linear optimal power flow (SCLOPF) [17] was subsequently solved for selected timesteps and a predefined set of N-1 line outage contingencies. AC power flow was then used for validation, confirming that the resulting operating state of the SecurEL reference system was consistent with that of the raw dataset.

### 4.4 Ambient-adjusted ratings (AAR)

The ampacity of overhead lines depends on conductor properties and weather conditions. The ratings in this dataset are calculated using the CIGRE TB 601 methodology [18], which provides a standardized thermal balance model for overhead conductors. Two datasets are provided, corresponding to different assumed maximum allowable conductor temperatures of 50 °C and 80 °C, respectively. These are AAR: capacity calculated for varying ambient temperature while other conditions are held at fixed reference values, as distinct from full dynamic line rating (DLR), which requires multi-variable weather monitoring, such as wind speed and direction.

Calculations are performed using open source software published by Statnett [19]. Line specific parameters are combined with discrete ambient temperature levels ranging from −30 °C to +30 °C in steps of 10 °C. A constant wind speed of 0.6 m/s is assumed, representing a standard industry reference condition, with wind direction set perpendicular to the conductor.

The ampacity calculations are based on a set of predefined parameters and assumptions regarding line characteristics, ambient conditions, and location. Key assumptions used in the calculations are summarized in Table 25, Table 26.

**Table 25:** Line related parameters and assumptions used in the ampacity calculations of AAR.

Parameter	Description / Value	Source
Conductor type and geometry, with electrical properties	As specified in *Handbook for grid planning*	[20]
Resistance at 50 °C and 80 °C	Calculated by the resistance at 20 °C from *Handbook for grid planning,* and the formula: $R\left(T\right)=R_{20}(1+\alpha \left(T-20\right)$ α=0.00403,temperature coefficient of aluminum	[7]
Maximum conductor temperature	50 °C and 80 °C	Assumption
Emissivity	0.8	Assumption
Absorption coefficient	0.8	Assumption
Line position	Stavanger, Norway	Assumption
Elevation above sea level	10 m	Assumption

**Table 26:** Ambient and environmental assumptions used in the ampacity calculations of AAR.

Parameter	Description / Value	Source
Air temperature	−30 °C to +30 °C in steps of 10 °C	Assumption
Wind speed and wind direction	0.6 m/s. Perpendicular to conductor	Industry standard value
Ground albedo	0.2	Assumption
Date time	03-10-25 10:00 (affecting solar irradiation)	Assumption
Clearness ratio	0.6 (dependent on cloud cover)	Assumption

### 4.5 Reliability data

Reliability data included in the dataset were based on national statistical data from the standardized Norwegian FASIT system, available through [10]. The data was filtered to include the years from 2015 to 2025 and was also filtered on component type and voltage level. National-average values were used instead of specific reliability data to avoid revealing the reliability of supply performance of the original grid.

### 4.6 Data provenance summary

Table 27 classifies the provenance, i.e. the origin, of each major dataset component: (i) measured/raw-derived, (ii) anonymized/transformed from measurements, (iii) synthetically generated, (iv) obtained from an external public source, or (v) based on an engineering assumption. Some components combine two categories where that more accurately reflects their actual data lineage (e.g. transformer parameters: a public standard type used as a substitution assumption).

**Table 27:** Provenance classification of the major dataset components.

Dataset component	Article table(s)	Provenance category	Note
Grid topology	Fig. 1, Table 3, Table 4, Table 5	(i) Measured/raw-derived	Retained from the original raw grid dataset
Branch electrical parameters	Table 5	(i) Measured/raw-derived	Retained from the original raw grid dataset
Branch/cable type designation	Table 6	(iv) External public source	Standard overhead-line/cable type parameters from the Handbook for grid planning [7]
Branch line lengths	Table 6	(iv) External public source	Estimated from NVE Atlas [11], a public database of Norwegian grid infrastructure to obtain realistic distances.
Transformer parameters	-	(v) Assumption	Standard 63 MVA 110/20 kV type substituted for the original, undisclosed transformer.
Load time series	Table 12	(ii) Anonymized/transformed	Derived from real SCADA measurements, anonymized as described in Section 5.2
Peak load prognosis	Table 14	(ii) Anonymized/transformed	Based on the DSO's real planning forecasts; released only as normalized factors.
Generator technical data	Table 7, Table 8	(i) Measured/raw-derived	Installed capacities and control mode reflect the real generator fleet.
Generation time series	Table 16	(iii) Synthetically generated	Produced by the PowerGAMA market-dispatch model [15], see Section 5.3
AAR	Table 20	(v) Assumption	Calculated from CIGRE TB 601 [18], see Section 5.4
Reliability data	Table 21	(iv) External public source	National-average statistics [9]
Temperature time series	Table 22	(iv) External public source	Weather station data [1] used as an anonymization substitute for the real grid location.
Standard line/cable type parameters	Table 9, Table 10	(iv) External public source	Handbook for grid planning [7].

## Limitations

This section is an attempt at a complete declaration of the limitations in this dataset.

A limitation of the generation dataset is that the generation time series are derived from an optimization model that excludes start-up and shut-down costs as well as detailed ramping constraints. This can result in dispatch patterns characterized by frequent transitions between zero output and high generation levels from one hour to the next. Although such behavior is also present in the raw dataset, it occurs more frequently in the modeled series, implying that statistical properties of the time series are not fully preserved. Consequently, the generation data should not be used in isolation for applications requiring realistic representations of hydropower operation.

A limitation of the load data concerns the load anonymization procedure (Section 5.2): as quantified there, the procedure systematically increases the ramp rate of the anonymized load series relative to the original (on average by a factor of 1.9) and degrades the weekly autocorrelation for a subset of load sites. Applications sensitive to high-frequency load variability or exact weekly demand patterns should be aware of this.

Reliability data (Table 21) are national-average failure statistics. This means they aren’t specific to this grid's components, geography or maintenance history.

The ambient-adjusted ratings (Section 5.4) are calculated for discrete ambient conditions, and therefore do not capture the full dynamic behaviour, for example wind-cooling effects, that a true dynamic line rating (DLR) would use. Applications requiring fully dynamic line rating behaviour are not directly supported.

The temperature time series (Table 22) is sourced from a weather station in Stavanger, a location chosen as a random substitute among stations with a complete hourly record for the study period. Since the true grid location cannot be disclosed, no closer comparison is stated.

In summary, applications directly supported by the released dataset include grid planning, contingency and reliability analysis, hosting-capacity assessment, and congestion or ambient-adjusted-rating studies at hourly resolution.

Applications requiring data or assumptions beyond what is released include: switching and network-reconfiguration or restoration studies, which require switch location and type data that is not included; studies requiring load-priority or criticality information, which is not included; detailed operational hydropower studies, since the synthetic generation series does not fully preserve real hydropower operational statistics; and studies requiring grid-specific reliability performance, or sub-hourly or non-steady-state behaviour.

## Ethics Statement

The authors confirm that they have read and comply with Elsevier’s ethical requirements for publication. This work does not involve human participants, animal experiments, or any data collected from social media platforms.

## CRediT Author Statement

**Susanne Sandell:** Conceptualization, Data curation, ​Methodology and software; **Sigurd Hofsmo Jakobsen:** Data curation, Methodology and software; **Astrid Lye Moum:** Data curation, software; **Daniel Bjerkehagen:** Methodology and software; **Harald Svendsen:** Methodology and software. All: Writing – Review and Editing.

## Declaration of Generative AI and AI-Assisted Technologies in the Manuscript Preparation Process

During the review of this work the authors used Claude Sonnet 5 (Anthropic), accessed through Claude Enterprise, in order to improve language and check for inconsistencies in the data descriptions. After using this tool, the authors reviewed and edited the content as needed and take full responsibility for the content of the published article.

## Footnotes

1 https://www.nve.no/energi/energisystem/vannkraft/oversikt-over-vannkraft/.
